# The barriers, benefits and training needs of clinicians delivering psychological therapy via video

**DOI:** 10.1017/S1352465821000187

**Published:** 2021-11

**Authors:** Joshua E. J. Buckman, Rob Saunders, Judy Leibowitz, Rebecca Minton

**Affiliations:** 1 Centre for Outcomes Research and Effectiveness (CORE), Research Department of Clinical, Educational & Health Psychology, University College London, 1–19 Torrington Place, London WC1E 7HB, UK; 2 iCope – Camden & Islington Psychological Therapies Services – Camden & Islington NHS Foundation Trust, St Pancras Hospital, London NW1 0PE, UK

**Keywords:** anxiety disorders, cognitive behavioural therapy, COVID-19, depression, psychological therapies, telemedicine

## Abstract

**Background:**

Due to the COVID-19 pandemic, mental health services have had to offer psychological therapy via video with little time to prepare or mitigate potential problems. Identifying the barriers, benefits and training needs highlighted by clinicians may support the effective delivery of care.

**Method:**

Changes in the mode therapy sessions were delivered in during 2020 were assessed in two high-volume psychological therapies services. Sixty-six therapists completed a survey about their experiences of delivering therapy via video.

**Results:**

The lockdown in March 2020 precipitated a dramatic shift from face-to-face to telephone and video-delivered sessions. Most clinicians (89%) found video-based sessions acceptable. Barriers to effective delivery included technological issues, problems with online platforms, and feeling more tired after sessions. Benefits included generalised learning from behavioural work, improvements in efficiency and in the therapeutic relationship, particularly in comparison with telephone-based sessions. Tutorials and support guides were recommended to maximise use of sessions via video.

**Conclusions:**

Video-delivered therapy was liked by clinicians and preferred to telephone-based sessions. Issues with platforms, internet connections and access for patients need addressing, local troubleshooting guides, video-based tutorials and greater support for low-intensity therapists to maximise uptake of video sessions where appropriate, may be beneficial.

## Introduction

COVID-19 has had an unprecedented impact on populations and healthcare services worldwide. Governmental responses to the pandemic including ‘lockdowns’ and quarantine rules have been associated with increases in loneliness and isolation (Bu *et al*., 2020) which in turn are associated with greater risk of developing mental health problems (Wang *et al*., 2018). General population surveys have shown rises in levels of depression and anxiety, and in the number of people with clinically significant mental illness (Shevlin *et al*., 2020). So, it is expected that the pandemic will lead to increased demand for psychological treatments for people with depression and anxiety (Holmes *et al*., 2020).

As national lockdowns were enforced across many countries, health services were required to move to remote modes of delivering care with little time to plan how to facilitate this change (Öngür *et al*., 2020). Some services may already have been delivering interventions remotely, but even for such services the requirement to do so for all treatment sessions will have required a considerable degree of change (Cromarty *et al*., 2020). This is likely to have had an impact on the experience of care for patients, and on clinicians’ experiences of delivering care. Understanding those experiences may be crucial to identifying and mitigating barriers faced by services in delivering the volume and quality of care required and expected of them (Greenhalgh *et al*., 2017), or in supporting their patients and clinicians to successfully access and deliver care (Patel *et al*., 2020).

Recent years have seen the development of the non-adoption, abandonment, scale-up, spread and sustainability framework (NASSS) to help healthcare organisations consider the process and likely adoption of novel technologies (Greenhalgh *et al*., 2017). The NASSS framework highlights the impact of the condition/illness for which a new technology is being introduced, the technology itself, the value of the technological change, those asked to adopt the technology, the organisation making such changes, and the wider context the changes are made within. The framework can help guide adoption projects that attempt to embed new technologies in health care settings, and guidelines on the competences required by therapists delivering care in such ways are outlined by the National Institute for Health and Care Excellence (Clark, 2011; National Collaborating Centre for Mental Health, 2010; National Institute for Health and Care Excellence, 2020; Richards *et al*., 2016; Roth and Pilling, 2007; Roth and Pilling, 2008). However, changes made by mental health services required to deliver the majority of care remotely, in the context of the COVID-19 pandemic, were seldom akin to a technology adoption project (Freeston *et al*., 2020). For some clinicians and patients this may have meant they needed to adopt technology that was new to them (e.g. for videoconferencing), but for others this will not have been the case (e.g. if conducting sessions over the telephone). Nonetheless, there may have been other changes made which might have focused on the structure of the services, the treatments offered, or the group of patients they were offered to (Cole *et al*., 2020).

Trials conducted in physical health settings have demonstrated high levels of satisfaction among clinicians and patients when care was delivered via video consultations, and equivalence of outcomes of face-to-face consultations (Car *et al*., 2020). Research comparing cognitive behaviour therapy (CBT) delivered face-to-face and via telephone has demonstrated similar levels of efficacy between intervention modes (Irvine *et al*., 2020; Mohr *et al*., 2012), and trials of internet-delivered (CBT) or video-delivered CBT have demonstrated better efficacy and cost-effectiveness compared with treatment as usual (Bower *et al*., 2013; Karyotaki *et al*., 2017; Kessler *et al*., 2009; Morriss *et al*., 2019). However, very few studies have investigated care delivered as-live via video and compared this with psychological therapy delivered face-to-face. The limited evidence from smaller studies comparing therapy delivered face-to-face and via video (Stubbings *et al*., 2013), or via telephone (Day and Schneider, 2002) suggests similar outcomes between modes. Yet, given the small sample sizes of these studies, concerns remain that less information is gained during video consultations and less still is gathered via telephone, that the media will affect the therapeutic relationship/alliance, and that a lack of access to appropriate hardware or technological issues may impact access to and the quality of patient care (Donaghy *et al*., 2019; Hammersley *et al*., 2019; Uscher-Pines *et al*., 2020). That notwithstanding, offering therapy via video or telephone can increase flexibility and reduce barriers to access related to the time required to attend sessions in-person, and due to stigma (McPherson *et al*., 2020). There has also been a body of practice-based evidence reviewed recently to guide services adapting to deliver care remotely due to the pandemic (Cromarty *et al*., 2020; Freeston *et al*., 2020). Furthermore, a recent review has shown that initial beliefs about the benefits of digitally delivered interventions for mental health problems, and the levels of personal support offered to users of these interventions, impacts upon their uptake (Patel *et al*., 2020). This may be particularly important to address the noted disparity in access to remote delivered psychological therapies, particularly by certain groups of patients that are less able to access or use technologies required to engage in remote delivered care (Eberly *et al*., 2020). As noted in the NASSS framework, the opinions and experiences of adopters/therapists asked to deliver care in such ways is crucial to the ability of services to be able to offer treatment in such ways (Greenhalgh *et al*., 2017).

Clinicians across healthcare disciplines often appear to have positive opinions about the use of video consultations. However, there has been very limited research exploring mental health professionals’ perspectives of delivering treatments via video, especially in response to the circumstances that have arisen as a result of the COVID-19 pandemic. We could only find one such study (Uscher-Pines *et al*., 2020); it was conducted with 20 psychiatrists in the USA, a number of whom were already using video-based consultations as part of their practice. That study found that most psychiatrists wanted to move back to in-person care, but they were largely positive about the transition to treatment via video. Several issues were highlighted though, including: patient-home disruptions; concerns about privacy; and a lack of internet access for more disadvantaged patients. The study did not address the uptake of therapy delivered in such a way among the clinical staff of different grades and levels of experience, and did not assess the barriers clinicians themselves faced in delivering video-based consultations. Identifying the perspective of clinicians about delivering care via video and any barriers they faced in doing so might help services consider how best to optimise treatment delivered in this way, and plan for the near future as remotely delivered therapy may be required, or either expected or requested by patients beyond the pandemic.

The aims of this study were: (1) to determine the changes in the way psychological therapy sessions were delivered during the year 2020 in response to the COVID-19 pandemic; (2) to ascertain clinicians’ overall experience, levels of confidence, and considerations of the acceptability in delivering therapy via video; and (3) to gain insights into the experiences of clinicians delivering treatment via video or telephone in terms of the barriers they have faced, the benefits they have noted, and the training needs or support they might require in order to more effectively deliver psychological therapies via video to adults with depression or anxiety disorders.

## Method

### Services and participants

This study explored the views of clinicians working in two Improving Access to Psychological Therapies services (IAPT) in London. IAPT services operate as primary care or community based psychological therapies services offering evidence-based psychological therapies to adults with common mental disorders such as depression or anxiety disorders (Clark, 2018). Data are collected at every patient contact including the purpose of the session, the mode by which therapy was delivered, and the date on which sessions were conducted, as mandated by NHS England (Clark, 2018). These data were used for all sessions with a purpose recorded as assessment, treatment, or a review session, during the calendar year 2020, in order to investigate changes in the mode of therapy delivery. In addition, a survey was created and sent to all 157 clinicians working in the two services. This included 47 low-intensity (LI) CBT therapists (also called Psychological Wellbeing Practitioners) and 110 high-intensity (HI) therapists (including Clinical Psychologists, Counselling Psychologists, and HI CBT therapists). LI therapists typically conduct approximately 60% of their consultations by telephone, and the remaining 40% are typically a mixture of face-to-face sessions and online typed ‘chat’ to support patients doing computerised or internet-based CBT. HI therapists typically conduct all of their sessions face-to-face.

During the initial stages of lockdown in England, there were a number of changes made to the platforms services could use to conduct sessions via video-conferencing. Some changes were the result of nationwide NHS policies due to concerns about data security, and others were due to newer platforms being made available, such as NHS Attend Anywhere, and a video platform embedded within the electronic patient record system used in the IAPT services.

### Measures

A survey (see Appendix A in Supplementary material) about experiences and opinions of delivered sessions via video was developed after consultation with clinicians of various grades/levels of seniority to support the wording and formatting of questions and of response options. The format of the survey, content and structure of questions and response options was informed by consultations with a working group of IAPT clinicians: the NHS Trust Chief Clinical Informatics Office, the video conferencing digital committee, a psychologist leading on digital innovation external to the IAPT services, and a quality improvement coach. Final details were agreed by the senior leadership team across the services.

### Procedure

The survey was sent to all clinical staff in the two services via email on 23 July 2020. In the email clinicians were asked to share their feedback on their usage of video sessions with patients to help understand what is working well, what the barriers are, and what support or training may be needed. A reminder email was sent two weeks later to encourage staff to complete the survey so the management team could understand any problems arising and how best to support the use of video-based therapy, with the closing date (7 August 2020) noted to staff.

### Analysis

**Figure 1.**
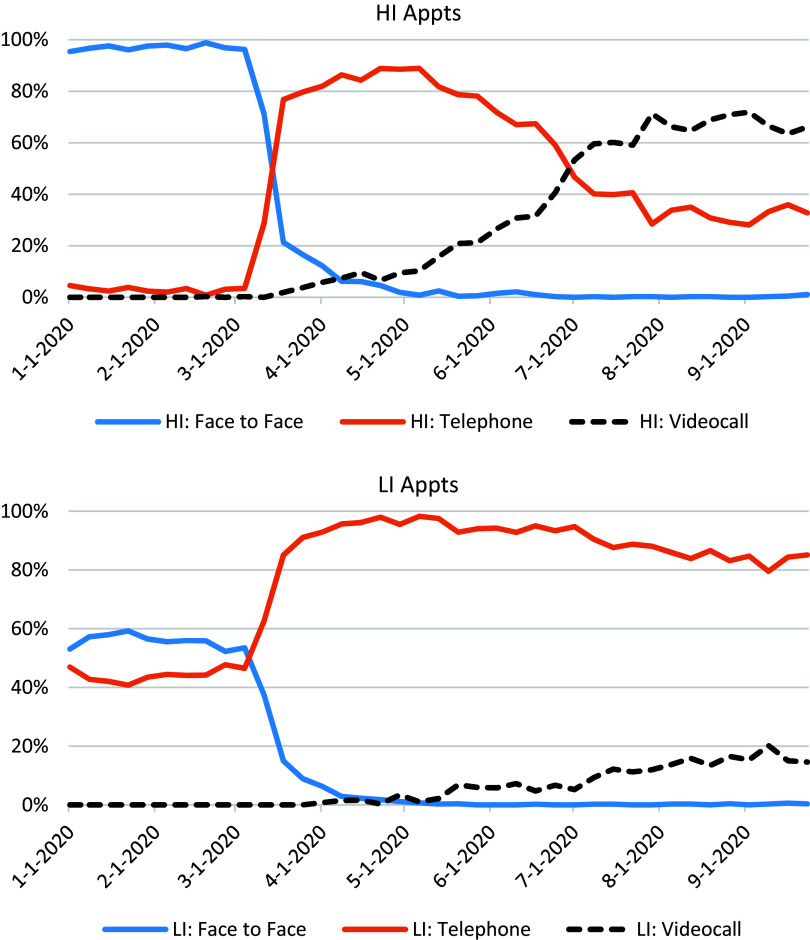
Trends in the use of face-to-face, telephone and video-therapy from January 2020 to June 2020, among HI and LI therapists.

In the first stage of analysis, trends in the media by which appointments were delivered during the weeks of 2020 starting in the first week of January up until 23 September 2020, were plotted in a graph (Fig. 1) to capture any changes. Separate plots were created for sessions conducted by HI and LI therapists.

Two non-clinical staff members collated all the survey responses. Responses were initially synthesised descriptively to compare the proportion of clinicians who had a positive experience of using video-therapy, their confidence in delivering video-therapy, and whether they considered video-therapy an appropriate way of delivering psychological therapies.

Another two members of the research team (J.E.J.B. and R.S.) then independently coded the qualitative data provided as feedback within the survey, and conducted a rapid thematic analysis highlighting initial themes and organising them into higher-order categories. Decisions on final themes were made by consensus and liaison with a third member of the research team (R.M.). Such rapid analyses are designed to provide information in rapidly changing situations such as was the case here (Uscher-Pines *et al*., 2020).

## Results

### Descriptive statistics

There were a total of 28,752 appointments in the study period, of which 49% were conducted by HI therapists, and 51% by LI therapists. Of all the appointments, 7249 (25%) were face-to-face, these were nearly exclusively conducted prior to the first national lockdown, and of these 62% were delivered by HI and 38% by LI therapists. There were also 16,624 (58%) telephone appointments (33% HI; 67% LI) and 4879(17%) via video (84% HI; 16% LI).

Sixty-six therapists completed the survey, this included 49 out of 110 (44.5%) HI therapists and 17 out of 47 LI therapists (36.2%). The number of video-therapy sessions delivered by clinicians at the date of survey completion was 2710.

### Trends in the use of video-therapy

Figure 1 presents the proportion of treatment appointments per week which were delivered face-to-face, by telephone, or by video, by HI therapists and LI therapists. In the first 10 weeks of 2020, the majority of all sessions were conducted face-to-face (including 97% of all HI sessions and 55% of all LI sessions), with approximately 44% of LI sessions and just 3% of HI sessions done over the telephone, and only two sessions done via video. However, at the start of national restrictions introduced to control the COVID-19 pandemic, in Week 11 of the year the proportion of sessions conducted over the telephone greatly increased while virtually no sessions were then conducted face-to-face. Conducting sessions via video was a rarely used option in the first few weeks of lockdown but became steadily more common throughout the successive weeks of 2020, such that by the beginning of July there were more HI sessions conducted via video (53%) than telephone (47%), and this trend continued into September when data collection for this study ended (66%). By contrast, very few LI sessions were conducted via video, with approximately 15% of sessions by 23 September; see Fig. 1 for details.

### Clinician experience of delivering video therapy

**Figure 2.**
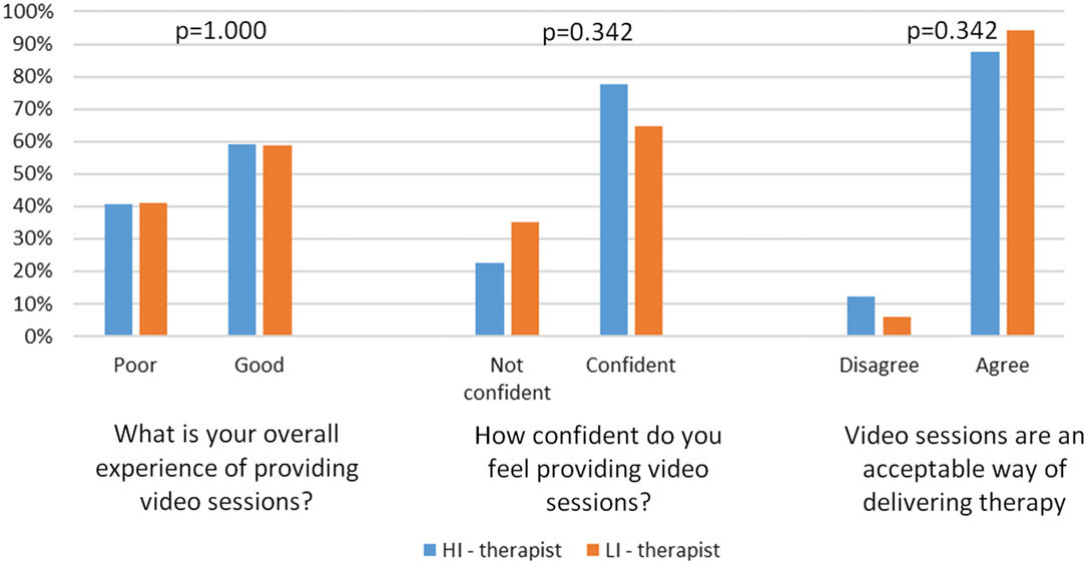
Proportion of clinicians endorsing statements on video treatment.

Fifty-nine per cent of both HI and LI therapists reported having a good or very good experience of delivering video sessions overall, with no evidence of a difference in the proportions of HI and LI therapists reporting this (Fisher’s exact test *p*-value=1.000); see the left-hand panel of Fig. 2. There was no evidence of a difference in the proportions of HI and LI therapists reporting that they were either confident or very confident about delivering video-based sessions (Fisher’s exact test *p*-value=0.342), although as shown in the middle panel of Fig. 2, confidence was slightly higher among HI therapists (78%) than LI therapists (65%). There was also no evidence of a difference in the proportions of HI (88%) and LI (94%) therapists agreeing that video sessions were an acceptable method of delivering treatment (Fisher’s exact test *p*-value=0.667); see the right-hand panel of Fig. 2.

### Problems and barriers to video-delivered sessions

The respondents noted a number of problems with delivering sessions via video. The most common issues raised were to do with poor internet connections (e.g. ‘…barrier is patients not having good enough Wi-Fi quality to sustain a good quality connection’ (HI 35)), lack of appropriate technology or hardware for either patients or clinicians (e.g. ‘Problems with tech [reliability of platforms and also device issues] have really impacted my ability to provide video sessions (HI 33)”), and in particular to do with the platforms used for video-therapy with 20 of the HI therapists (41%) and seven (41%) of the LI therapists highlighting such issues. It was this latter category which elicited the some of the most strongly worded responses too, for example ‘Please can we all use zoom again!!’ (HI 26) and ‘Platforms not working properly [freezing, delays between picture and sound or being temporarily unavailable] is one of my biggest stresses currently’ (HI 20); see Table 1 for details. Four HI therapists commented that a number of patients seemed not to want sessions via video (e.g. ‘The biggest barrier is patients not wanting to use it’ (HI 35)). Two HI therapists also reported that they were put off or otherwise not keen to conduct sessions over video (e.g. ‘I’ve not used Attend Anywhere as avoidant of another log in/link to set up and the feedback about its unreliability’(HI 32)).

**Table 1.** Themes and illustrative quotes of problems with video-therapy identified by respondents

Themes	Sub-themes	Illustrative quotes
**Technological issues**	**General technological issues**	When the technology works they are far superior to telephone alone (HI 3)
		When it works it is convenient and nice to see clients face to face instead of just hearing them (HI 4)
		Quick and easy when the technology is working (HI 22)
		Issues for me are more related to unreliable technology (HI 2)
		I’m happy using video, it’s the technology that is an issue (HI 3)
		No support – just better IT functionality and efficiency are needed (HI 49)
		It has been a generally difficult time IT-wise and this tends to overshadow the video experience (HI 6)
	**Platform-related issues**	The quality of the platform makes a huge difference. I am using Zoom for non-work related activities and the quality of the audio and video is vastly superior, as is, the reliability of the connection (HI 35)
		When Attend Anywhere works, it’s really good (HI 23)
		I arranged for one patient to do a video call via IAPTus (instead of AA) as an experiment, but when I clicked on the link to start the session it didn’t work. I really don’t know why but it has put me off of using it (HI 35)
		I feel confident using the platforms, Microsoft Teams is good but I switched to it after Attend Anywhere failed a few times (HI 4)
		Our current platforms have no whiteboard and I can’t upload a Word document on Attend Anywhere (HI 7)
		Use of Zoom (would improve things) (HI 19)
		No training needed – just better platforms (HI 20)
		I’d just like a better platform. Have tried all of them and none of them are very good quality. I would like to use Zoom, or have a platform that has the same excellent quality (HI 36)
		I don’t require any training. As long as the technology works and the software doesn’t have audio- or video issues (like AA often has) I don’t see a problem with using video calls for therapy (HI 30)
		…We need better platforms though (HI 29)
		I think just a more reliable video platform (LI 10)
		One platform, reliable and high quality please – not constant interference and fake concern re data protection (LI 11)
		I have found Attend Anywhere is not stable as MS Teams and would advocate moving to Teams (HI 1)
		Can we use Zoom, I’ve used this for private practice and other meetings and its functionality and reliability are good (HI 32)
		Bring back Zoom! (HI 3)
		Attend Anywhere can be glitchy or doesn’t work at all with some clients, where they are in the waiting room and I can’t see them or it keeps crashing. Teams is more reliable for me, but I have heard about others finding it tricky (HI 4)
		Is it possible to have a platform whereby you can work on an active document and this is saved on both ends of the call (therapist and patient)? (HI 11)
		When sharing video feedback with clients I have to send it for them to view separately due to problems with buffering when sharing in Attend Anywhere (HI 5)
		Platforms not working properly (freezing, delays between picture and sound or being temporarily unavailable) is one of my biggest stresses currently (HI 20)
		Zoom is much easier to use as a platform (LI 15)
		Having a solid and stable platform to use would increase my confidence in using video (LI 16)
		Nothing has worked as well as Zoom unfortunately, although IAPTus is thankfully better than AA (HI 20)
		AA often has a few seconds delay in the video that makes sessions difficult to follow. It has happened with several clients and it is quite tricky to have a conversation, which has at times meant that I had to call them whilst keeping the video on (HI 29)
		Bring back Zoom! (LI 3)
		Please can we all use Zoom again!! (HI 26)
		I’m less sure about the IG implications for using Teams with patients and not sure how we practise using IAPTUS because it only seems to generate a link with genuine patients? Thank you! (LI 5)
	**Problems with internet connection or lack of device for patient or therapist**	…barrier is patients not having good enough Wi-Fi quality to sustain a good quality connection (HI 35)
		The main issue is about connection and the disconnection of the videos, not being able to access it (HI 24)
		The internet connection/system connection is the main issue, and teaching clients how to use it and prepare for sessions, and attend them on time (HI 7)
		Often IT/connection issues on both ends (HI 11)
		Currently most of my clients don’t have the ability to use video so not an option anyway; just more change! (HI 44)
		Generally positive use of video, however had cut out or not worked at times and this can be very difficult when discussing sensitive topics and can disrupt the flow of the session and takes longer to do sessions if this happens (HI 21)
		Overall, the video platforms keep failing due to connection issues and images or audio can be blurred at times (HI 34)
		Problems with tech (reliability of platforms and also device issues) have really impacted my ability to provide video sessions (HI 33)
		It’s not the training or the technology that’s at fault, it’s the poor quality and unreliability of the internet connection between therapist and patient. Physical separation is only increased by trying to share your fears to a pixelated face. I can work with patients without ever seeing how they move or engage in real life but I find the connection issues almost unbearable (HI 14)
		Currently sorting with IT, may be able to offer soon (HI 42)
		Connection can sometimes be poor which is difficult (HI 7)
		What would be great is a reliable video system, and good Wi-Fi connection from both sides would be great. If the system would be able to also pick up slower speed Wi-Fi would be great (HI 24)
		My patient’s internet lost bandwidth during the session this morning, and we just switched to video on our phones – it was absolutely seamless. I do have good internet connection at home and of course, this makes all the difference (HI 28)
		In general, it has been okay but my experience with Attend Anywhere is that it’s very laggy and the audio/visual often do not match up which is very disconcerting! (LI 7)
**Desire to use video**	**Patient not wanting video sessions**	The biggest barrier is patients not wanting to use it (HI 35)
		I have had many patients decline this option however, more than I would have anticipated, with many preferring the phone (HI 41)
		In fact, it has been fine working with video on the whole. I have only had one patient refuse it (HI 6)
		I have discussed with my peers about how I offer a video sessions to clients, and they have been surprised as they do it differently. They have been offering videos as the first choice and had an expectation that the client would prefer that. In contrast, I have asked clients whether they prefer a telephone or video, and they almost or prefer telephone (HI 47)
	**Therapist not wanting to conduct video sessions**	I’ve not used Attend Anywhere as avoidant of another log in/link to set up and the feedback about its unreliability (HI 32)
		I had good intentions and was keen to use but have ended up having to switch to phone in the majority of my sessions (HI 33)
	**Miss face-to-face contact**	I also do miss being in the room with patients and being able to do active therapy e.g. leave the room to do an experiment (HI 17)
	**Too busy to learn to use video functions properly**	No time to just get myself set up for this as I’m just so overwhelmed with other things (LI 5)
		Trying to work out another platform with Attend Anywhere just feels like something extra I don’t have the headspace for (HI 44)
**Physical or environmental issues**	**Patient or clinician lacking private, safe or quite space for therapy**	As long as there are no connection issues or environmental issues (confidential spaces to speak etc.) I prefer using video sessions to face to face! (HI 25)
		Also my home has noisy works happening next door. Callers also not always able to hear me in Teams meetings which makes me anxious it won’t be reliable with client sessions. I don’t have a private room so Bluetooth headphones are needed to have privacy of what is being discussed (HI 32)
		Working from home with children in the house makes it slightly more tricky to consider video sessions for fear of interruption/noise levels (HI 41)
	**Video sessions more tiring**	It is also just so much more draining mentally, and cannot be good for our eyes if we see all our patients via video (HI 11)
		I think it would be helpful to reduce contacts if more appointments will be video – it is much more exhausting (HI 43)
		It takes up so much time and then I am completely drained after a video session (HI 11)
		I do notice that doing lots of video sessions is more tiring than doing the same amount of face to face work. So e.g. 4–5 face to face sessions in day is achievable, it’s a bit more of a struggle with video sessions – you just feel more fatigued. It also strains your eyes and your voice more to do them (HI 15)
		I am happy to do video sessions and prefer them to telephone as it is more similar to face to face therapy. I do find it more draining than face to face (HI 20)
	**Clinician finding it more stressful**	I’m generally not a techy person and so having to sort out all this stuff remotely/on my own has been one of the worst things about the whole move to working from home. It’s just added stress on top of an already stressful situation! (HI 44)
		Having very intense conversations about difficult material is a regular part of being a therapist and it is very stressful when the platform doesn’t work properly during these conversations (e.g. trying to check risk or discuss childhood trauma but having to ask the person to repeat themselves because the screen freezes) (HI 20)
		Although it’s positive to have some choice, I have felt a bit overwhelmed about having to learn to use different platforms (LI 5)
**Treatment related issues**	**Can’t use a whiteboard**	Sharing whiteboards and allowing patient to also draw on whiteboard and drawing out formulations and other types of diagrams which has often taken too long as didn’t go smoothly so wasted some time in session on this (HI 18)
		Other main issue is it’s hard to work through things together on a whiteboard or similar (HI 7)
		I also do miss being able to draw something out on a whiteboard (HI 17)
	**Treatment more difficult (including** ***in vivo*** **work)**	…Effectively and confidently introducing stooges. I have done this a few times successfully and a few times less successfully! (HI 6)
		How to do the social anxiety stooge experiment over video call (HI 26)
		It is frustrating not being able to record the sessions easily e.g. for social anxiety behavioural experiments (HI 5)
		The disadvantage of video sessions is that the videos of the patient are not always that clear so it’s hard to see their emotional expression, also just not being able to be in the room with them, we almost definitely miss some information e.g. body language, use of Safety Behaviours in session and sharing documents on line often takes longer. Overall though I would say it’s ok and much less problematic than I would have anticipated (HI 18)
	**Less efficient**	I have to spend at time 2–3 sessions just to teach them how to use it, how to complete questionnaire, setting boundaries so they treat it as a therapy session and respect it (HI 24)
		Sharing whiteboards and allowing patient to also draw on whiteboard and drawing out formulations and other types of diagrams which has often taken too long as didn’t go smoothly so wasted some time in session on this (HI 18)
		I think our contacts need to be adjusted/reduced as we are spending so much additional time preparing for and saving and sending documents to clients after sessions (HI 11)
		My workload has increased a lot due to emailing formulations, material, video call invites (HI 11)
		I think clients should be given extra 2 sessions on top of their treatment because of IT issues (HI 24)
	**Face-to-face is better**	The appropriateness depends on the client’s circumstances, the clinical presentation and whether or not there is a pandemic going on! Face to face work is always going to be more appropriate for talking therapies unless there are issues for the client accessing onsite sessions (HI 13)
		I do think there are some patients who have struggled to engage with the therapy over video and think that they would have benefited more if we had physically seen each other (HI 17)
		Face to face is preferable but video works too if done well and there is less connection and system interruption (HI 24)

Away from problems related to platforms and connectivity, one LI and one HI therapist noted that they were too busy to learn how to skilfully deliver sessions via video (e.g. ‘No time to just get myself set up for this as I’m just so overwhelmed with other things’ (LI 5)). Three HI therapists highlighted issues to do with finding a quiet, safe, or private space either for themselves or their patients, in order to conduct video sessions (e.g. ‘Working from home with children in the house makes it slightly more tricky to consider video sessions for fear of interruption/noise levels’ (HI 41)). In addition, four HI therapists noted that they found delivering sessions via video more tiring, draining, or a strain on the eyes, compared with delivering sessions face-to-face (e.g. ‘It takes up so much time and then I am completely drained after a video session’ (HI 11)). Three others reported that therapy was more difficult due to a lack of physical or shared resources, including a whiteboard (e.g. ‘Sharing whiteboards and allowing patient to also draw on whiteboard and drawing out formulations and other types of diagrams which has often taken too long as didn’t go smoothly so wasted some time in session on this’ (HI 18)). Four HI therapists noted that it was more difficult to conduct behavioural or *in vivo* work with patients over video, particularly stooge experiments for patients with social phobia (e.g. ‘It is frustrating not being able to record the sessions easily, e.g. for social anxiety behavioural experiments’ (HI 5)). Five HI therapist reported that video-based sessions were less efficient, partly due to technological difficulties (e.g. ‘I have to spend time at 2–3 sessions just to teach them how to use it, how to complete questionnaire, setting boundaries so they treat it as a therapy session and respect it’ (HI 24)), and partly due to the additional time taken to provide patients with the materials they would ordinarily share face-to-face (e.g. ‘My workload has increased a lot due to emailing formulations, material, video call invites’ (HI 11)). There was also a general sense from a small number of respondents that they find face-to-face sessions more effective and personally more enjoyable (e.g. ‘I do think there are some patients who have struggled to engage with the therapy over video and think that they would have benefited more if we had physically seen each other’ (HI 17) and ‘I also do miss being in the room with patients and being able to do active therapy, e.g. leave the room to do an experiment’ (HI 17)). Table 1 has a full breakdown of the themes and sub-themes, with quotes to illustrate each of them.

### Benefits to video-delivered sessions

The respondents also noted a number of benefits of video-based sessions, some with very strongly worded comments of support for this way of working, and some of those were the same respondents that also noted a number of barriers listed above (see Table 2 for a full list of quotes). Eight HI therapists and two LI therapists reported that video sessions were more accessible for patients (e.g. ‘Easy access for clients compared to attending face to face’ (HI 27) and ‘Accessibility it provides, both regarding times and comfort (e.g. people with [long-term conditions] LTCs can sit on comfortable chairs at home)’ (LI 8)), that they have brought greater flexibility for patients and clinicians (e.g. ‘I love the convenience of being able to work remotely with patients by video…. Use of video gives me more flexibility about when I offer sessions’ (HI 35) and ‘Flexibility for the patient as well as clinician’ (HI 6)), and there was a suggestion that using video sessions has helped improve attendance rates (e.g. ‘Less cancellations I think’ (HI 7)). Six HI therapists commented that offering video sessions has helped improve their work–life balance (e.g. ‘I am better able to meet my patient’s needs while also managing my own work–life balance. I would like to do more video work in the future’ (HI 35)), or that they prefer working from home and offering video sessions (e.g. ‘Being able to work from home – allows more focus on patient, less distraction and stress of being in the office’ (HI 40)).

**Table 2.** Themes and illustrative quotes of benefits of video-therapy identified by respondents

Themes	Sub-themes	Illustrative quotes
**Flexibility and accessibility**	**More accessible for patients**	Easier for patients to access this at home (HI 21)
		More convenient for patients (HI 14)
		Making therapy more accessible for patients (HI 36)
		Easy for patients to access (HI 15)
		Quick and easy when the technology is working (HI 22)
		Accessibility for the patient (HI 23)
		Easy access for clients compared to attending face to face (HI 27)
		Also extremely convenient for therapist and patient (HI 18)
		Accessibility it provides, both regarding times and comfort (e.g. people with LTCs can sit on comfortable chairs at home) (LI 8)
		Offers choice to clients who prefer it (LI 12)
		Gives patient the option when f2f isn’t available (LI 3)
		Face to face work is always going to be more appropriate for talking therapies unless there are issues for the client accessing onsite sessions (HI 13)
		Easier access for some clients (HI 8)
	**Better attendance**	Less DNAs (HI 15)
		Less cancellations I think (HI 7)
		Better attendance in some instances (HI 8)
	**Flexibility for patient or clinician**	Flexibility for the patient as well as clinician (HI 6)
		Patients can be more flexible in attending as no travel time (HI 7)
		I have been able to offer clients the choice of phone or video. And then been able to explore with them why they have chosen one of the other (HI 48)
		It makes times of sessions more flexible because we are not limited by room space, but we can still see the patient and their non-verbal reactions (HI 30)
		I love the convenience of being able to work remotely with patients by video … Use of video gives me more flexibility about when I offer sessions (HI 35)
		When it works it is convenient (HI 4)
		Greater flexibility for me as therapist (HI 23)
		Convenient for clients (and therapist) (HI 29)
		Doing it from own home, no risk of infection (LI 11)
**Home life**	**Better work–life balance**	I am better able to meet my patient’s needs while also managing my own work life balance. I would like to do more video work in the future (HI 35)
		No travel time/cost (LI 11)
	**Prefer working at home**	My set-up at home is better than it is in the office so I feel more equipped to deliver therapy with all the resources I need at my disposal (HI 35)
		Being able to work from home (HI 9)
		Nice to be able to work from home (HI 17)
		Being able to work from home – allows more focus on patient, less distraction and stress of being in the office (HI 40)
**Effectiveness**	**Behavioural work better/taught skills more generalisable if in their home**	Being ‘invited’ into clients’ homes means that the active work can feel more real and perhaps generalise more easily (HI 1)
		I have done many in-session experiments that would not have been possible without video calls, e.g. doing contamination experiments in the client’s environment (HI 30)
		Has been helpful for certain patients to be able to do behavioural experiments in their home (HI 17)
		Being able to see patients in their own home which can sometimes be really useful in terms of them being able to carry out experiments in their real environment while being there on video to support them. Also extremely convenient for therapist and patient (HI 18)
	**As effective or nearly as effective as face to face**	Closest thing to f2f appointments (HI 25)
		Video makes it more similar to face to face appointments (HI 43)
		Realising it is as effective as face to face (HI 12)
		They have been an acceptable alternative to face to face. Obviously different to face to face, but good enough (HI 48)
		Being able to see the patient and that they can see me too, feels as close to a face to face session as possible (LI 9)
	**Like using video/it went well**	I go between IAPTus (very convenient from admin POV) and Attend Anywhere – when AA works, it’s really good. I think [our service] has actually improved as a service in the last 6 months: video work has played a significant role in this (HI 23)
		As long as there are no connection issues or environmental issues (confidential spaces to speak etc) I prefer using video sessions to face to face! (HI 25)
		IAPTus Video is great – really easy to use, very happy with it! (LI 1)
		When it works it is convenient and nice to see clients face to face instead of just hearing them (HI 4)
		I love doing video work and would appreciate more opportunities to work this way (HI 35)
		Generally positive use of video (HI 21)
		I liked it – IT was very smooth, didn’t cut out (LI 15)
		I think I feel quite confident in providing video sessions now (HI 15)
**Practical benefits**	**Screen prompts/note access**	Easier to look at notes of previous sessions and make notes without it taking focus away from interaction with patient (LI 8)
		Been able to have prompts not visible to patient (HI 7)
		Can have session prompts/preparation up on the screen to refer to if doing interventions which are less familiar (HI 25)
	**Efficient use of time**	I also spend less time travelling (HI 35)
		Can make notes in real time (saving admin time) (HI 25)
		Less time in day spent travelling to different locations, e.g. GP surgery (HI 15)
	**Recordings**	Recordings which you can share (HI 6)
	**More easily share online resources**	Being able to share online materials and formulations with clients easily (HI 2)
		Ability to share screen to incorporate materials (HI 25)
		It’s really easy to share/access materials with patients on the screen and by email afterwards (HI 28)
	**No room pressure**	I don’t have to deal with the constant headache of booking rooms (HI 35)
		No room pressure (HI 7)
**Advantages over telephone-based sessions**	**Better than phone in general**	Better to have the visual share screen otherwise difficult to do over phone (HI 21)
		I am happy to do video sessions and prefer them to telephone as it is more similar to face to face therapy (HI 20)
		When the technology works they are far superior to telephone alone (HI 3)
		This is the best platform after F2F (HI 19)
		It’s better than the phone (HI 16)
	**Important to see the client**	Getting to see the client and their reactions (LI 10)
		The ability to interact with others in a different way (LI 16)
		The client could see my face which put them at ease (LI 17)
		Being able to see the patient and them being able to see me (LI 4)
		Nice to see clients face to face instead of just hearing them (HI 4)
		In comparison to telephone sessions, more helpful in seeing the client (HI 39)
		Being able to see the client (HI 19)
		It has been nice to see the client and simulate a normal face to face therapy session (HI 46)
	**Better therapeutic relationship (vs telephone)**	Find it easy to build rapport if I can see the person, especially with PTSD work (HI 31)
		So much more interactive than phone work. Much more connected to client (LI 1)
		It’s good for clinician and patient to see each other for a better connection and to be able to read facial expressions (LI 7)
		Good to see patient’s face – can help with rapport. Can also use non-verbal communication which is helpful in therapeutic relationship (LI 5)
		You can see the client and connect more (HI 32)
		Being able to read the situation with client and use skills in as things come up, i.e. empathy (HI 34)
		You can see the client and develop more of a rapport than you would over the telephone (HI 5)
		It’s more personable and can help with the interpersonal relationship and rapport building (HI 38)
		Being able to see the patient and have non-verbal communication (HI 20)
		I can read body language and feel more of a rapport with someone (HI 10)
		Better than telephone I find in terms of building rapport (HI 8)
		Non-verbal cues and body language can be seen which can help build therapeutic trust and rapport (HI 11)
		You feel more connected to the client (HI 26)
		You can see the person, see their face and get to know another aspect of them (LI 2)
		I think it’s quite a personal and engaging kind of conversation, given that the technology doesn’t go ker-spling (HI 28)
	**More information gained than over the phone**	On the phone you can miss the non-verbal cues, so I have found it able to pick up lot of subtle things patient cannot voice sometimes (HI 34)
		This also helps therapist identify any avoidance or physical therapy interfering behaviours (HI 11)
		Seeing the client provided more information than just speaking to them on the phone (HI 44)
		Observing and making use of non-verbals (HI 47)
		Easier to evaluate non-verbal communication (HI 49)
	**Communicate better (vs telephone), include share screens**	Better communication (HI 21)
		Ability to share screen where possible (HI 33)
		Reviewing homework tasks together with greater ease (HI 2)
		Bring in therapy materials both audio/visual and written word (HI 23)
		Can share your screen (HI 26)
		Find them easier than telephone for lots of things e.g. sharing formulations (HI 27)
		I also find screen sharing very useful (HI 30)
		Materials can be used/shared via screen (LI 8)
	**Facilitates treatment type specific skills**	Sessions seem more CBT focused (HI 25)
		Being able to do EMDR (HI 20)
		Being able to share CBT diagrams (HI 2)
	**Behavioural work better**	Can do more behavioural things in sessions (HI 21)
		Better for carrying behavioural experiments (HI 39)
		This can be good for learning and for behavioural experiments (HI 6)
	**More engagement (vs telephone)**	Better engagement (HI 39)
		Being able to see client, better engagement (HI 41)

There were many comments on the effectiveness of delivering therapy via video-sessions, with 13 HI therapists and three LI therapists commenting on this (e.g. ‘Being “invited” into clients’ homes means that the active work can feel more real and perhaps generalise more easily’ (HI 1), ‘Realising it is as effective as face to face’ (HI 12) and ‘I love doing video work and would appreciate more opportunities to work this way’ (HI 35)). There were a number of practical benefits noted by respondents, including being able to use screen prompts or access notes live in session (e.g. ‘Easier to look at notes of previous sessions and make notes without it taking focus away from interaction with patient’ (LI 8)), improvements in efficiency (e.g. ‘Less time in day spent travelling to different locations, e.g. GP surgery’ (HI 15)), being able to make shareable recordings of the sessions (HI 6), having easier access to shareable online resources (e.g. ‘It’s really easy to share/access materials with patients on the screen and by email afterwards’ (HI 28)), and not having to find/book clinical rooms which can be difficult for face-to-face sessions (e.g. ‘I don’t have to deal with the constant headache of booking rooms’ (HI 35)).

Some of the most common comments overall highlighted the ways in which video-based sessions are preferable to telephone-based sessions, with 19 HI therapists and eight LI therapists providing such comments. In particular, respondents highlighted the benefits to the therapeutic relationship (e.g. ‘Good to see patient’s face – can help with rapport. Can also use non-verbal communication which is helpful in therapeutic relationship’ (LI 5) and ‘So much more interactive than phone work. Much more connected to client’ (LI 1)), the improvement in information that can be gathered when doing video sessions (e.g. ‘On the phone you can miss the non-verbal cues, so I have found it able to pick up lot of subtle things patient cannot voice sometimes’ (HI 34) and ‘This also helps therapist identify any avoidance or physical therapy interfering behaviours’ (HI 11)), that they find it important to see their clients (e.g. ‘In comparison to telephone sessions, more helpful in seeing the client’ (HI 39) and ‘The client could see my face which put them at ease’ (LI 17)), and find that they can communicate better via video (e.g. ‘Find them easier than telephone for lots of things, e.g. sharing formulations’ (HI 27)). In addition, respondents noted a number of ways in which video-based sessions helped facilitate treatment more effectively than sessions conducted over the telephone. These included the use of treatment type specific skills (e.g. ‘Being able to do EMDR’ (HI 20)), behavioural work being better (e.g. ‘Better for carrying behavioural experiments’ (HI 39)) and better engagement from clients (e.g. ‘Being able to see client, better engagement’ (HI 41)).

### Training needs and support

In the survey sent to clinicians we asked for opinions on any training or practical support that would aid in the delivery of video-based sessions. Respondents highlighted several areas of training and ways in which training might be delivered for them, including clinical support, for example with behavioural work (e.g. ‘How to do the social anxiety stooge experiment over video call’ (HI 26)), training on the functions of the platforms used for video sessions (e.g. ‘One to one tutorial on uploading documents whilst in sessions, screen sharing, etc. as differs from platform to platform and fear that whilst doing these I will cut off the call’ (HI 37)), and two HI therapists suggested they would like training on the research evidence for the effectiveness of video-based therapy (e.g. ‘Summary of research evidence on digital therapy’ (HI 13)). However, eight HI therapists noted that they did not require any further training (e.g. ‘At the risk of sounding smug, I think I’m ok’ (HI 28) and ‘I have had ample online training’ (HI 9)).

One HI therapist proposed that training for clients on how to set up and use video sessions would be useful (e.g. ‘A video for clients and guidance how to use sessions, and attend them on time. 80% of clients are ok but 20% struggle and we can spend a lot of time on dealing with how to use it, how to attend on time, finding a system that works for them and the connection works’ (HI 24)). In addition, 14 HI therapists and six LI therapists commented on the ways in which training or support might be most usefully provided. These included troubleshooting guides of ‘top tips’ (e.g. ‘Troubleshooting library for when, for example, there is no sound on a video you are playing through screen share’ (HI 1)), video tutorials (e.g. ‘Video clips would be helpful in perhaps setting up audio and visual displays before calls so that your face is clear, and no issues with audio during the session’ (HI 34)), and practical support or sessions with information and technology based staff within the organisation (e.g. ‘Maybe just some expert tips of lesser known features of the platform. Or tips about how to make the most of shared screen et cetera’ (HI 47)). Finally, four HI therapists and one LI therapist commented that the time taken to engage in training is a concern (e.g. ‘Although it’s positive to have some choice, I have felt a bit overwhelmed about having to learn to use different platforms’ (LI 5)) and that they would like time to role play using the platforms (e.g. ‘Having a colleague(s) who linked with to practice using together’ (HI 44)). See Table 3 for a full list of quotes for each theme and subtheme.

**Table 3.** Themes and illustrative quotes of training needs for video-therapy identified by respondents

Themes	Sub-themes	Illustrative quotes
**Training for clinicians**	**Clinical support (experiments, formulations)**	How to set up experiments (HI 21)
		Guidance docs re: top tips for working clinically, e.g. editable formulations (HI 33)
		Effectively and confidently introducing stooges. I have done this a few times successfully and a few times less successfully! (HI 6)
		I would appreciate training on what I could use to do formulations and use a whiteboard (HI 7)
		Sharing whiteboards and allowing patient to also draw on whiteboard and drawing out formulations and other types of diagrams which has often taken too long as didn’t go smoothly (HI 18)
		How to share documents in real time so that we each have a copy of updated formulation. Also, having a shared folder with blank formulations and other material that is useful for delivering sessions remotely would be helpful (HI 11)
		How to do the social anxiety stooge experiment over video call (HI 26)
		Some guidance about how best to use materials online e.g. filling in vicious cycle, is there a better techy way to do this? (LI 9)
		I would like to explore options for using a graphic tablet so that I can draw out formulations by hand and share them on the screen with my patients (HI 35)
		It’s hard to work through things together on a whiteboard or similar (HI 7)
		Could we have some guidance on how to do Step 3 via telephone for presentations that require F2F e.g. social anxiety? (HI 10)
	**Learning about research evidence for effectiveness**	I am curious about how clients and I use video. What is different, in a positive way and in a less than positive way. I am interested in the process (HI 48)
		Summary of research evidence on digital therapy (HI 13)
	**Training on functions of video platforms, and differences between platforms**	A video library of how to do things like share screens (HI 1)
		Ways of writing and drawing in session so both can see (HI 13)
		Guidance around use and forms to send client for each platform (HI 31)
		If training was to be provided then helpful to have some video clips that can be easily accessed on using features that might be less used because of lack of familiarity etc., e.g. whiteboards (HI 2)
		How to video record your screen on the Trust laptop while using global protect (HI 14)
		One to one tutorial on uploading documents whilst in sessions, screen sharing etc. as differs from platform to platform and fear that whilst doing these I will cut off the call (HI 37)
		Maybe just some expert tips of lesser-known features of the platform. Or tips about how to make the most of shared screen et cetera (HI 47)
		Using share screen to draw formulations/white board type of programmes? Resources on that? (HI 12)
	**Had enough training on this already**	I have found the training sessions by OXCADAT/BABCP helpful for specific advice about providing interventions remotely (HI 27)
		I don’t require any training. As long as the technology works and the software doesn’t have audio- or video issues (like AA often has) I don’t see a problem with using video calls for therapy (HI 30)
		At the risk of sounding smug, I think I’m ok (HI 28)
		No support/training needed (HI 25)
		I feel confident using the platforms (HI 4)
		I think I feel quite confident in providing video sessions now (HI 15)
		I don’t need training (HI 16)
		I have had ample online training (HI 9)
**Training for patients**	**How to use platforms**	Some clients need more IT support from the clinicians. I have to spend at time 2-3 sessions just to teach them how to use it (HI 24)
	**Basic IT skills**	A video for clients and guidance how to use sessions, and attend them on time. 80% of clients are ok but 20% struggle and we can spend a lot of time on dealing with how to use it, how to attend on time, finding a system that works for them and the connection works (HI 24)
**IT support**	**Top tips or troubleshooting documents**	Troubleshooting library for when, for example, there is no sound on a video you are playing through screen share (HI 1)
		Guidance documents might be useful for reference (LI 16)
		Guidance docs (HI 21)
		Guidance docs (HI 33)
		Guidance documents (LI 13)
		I think guidance documents would also be useful (HI 46)
		To know how to test and be able to set these up before sessions. General top tips of best way to use video platforms i.e. camera angles, lighting, test audio and also adjust things if need to during the call (HI 34)
	**Video clips with walk-through guides**	How to manage technical difficulties e.g. cutting out or freezing – video clips, one to one tutorial (HI 21)
		Video clips would be helpful in perhaps setting up audio and visual displays before calls so that your face is clear, and no issues with audio during the session (HI 34)
		Helpful to have some video clips that can be easily accessed (HI 2)
		Video clips! (LI 7)
		Video clips (HI 21)
		Video clips are useful as you can watch them in your own time (HI 31)
		Video clips and documents (HI 3)
		Video clips, guidance documents (LI 8)
		Tutorial or video clips (LI 4)
	**Practical support/demonstrations/guidance/drop-in sessions (e.g. screen share)**	Drop-ins with IT (HI 21)
		Drop-in sessions (HI 40)
		I would like someone to demo how to use IAPTus video (HI 35)
		One to one tutorial on uploading documents whilst in sessions, screen sharing etc. as differs from platform to platform and fear that whilst doing these I will cut off the call (HI 37)
		Drop-in sessions or video demonstrations of video platforms on IAPTus and attend anywhere and also teams (HI 40)
		IT support (HI 42)
		One to one on expanding use of 365 around video work (HI 23)
		One-to-one tutorial (LI 12)
		One-to-one tutorial (HI 18)
		Tutorials (HI 11)
		Drop-in sessions and guidance docs (HI 46)
**Time and time pressures**	**Time to familiarise with platforms or their functions**	Given some time that is specifically set aside for getting set up with video work (HI 44)
		Support from managers in terms of caseload to allow time to learn all the different modalities (HI 38)
		Although it’s positive to have some choice, I have felt a bit overwhelmed about having to learn to use different platforms (LI 5)
	**Time to practise/role-play**	Having a colleague(s) who linked with to practice using together (HI 44)
		Not sure how we practise using IAPTUS because it only seems to generate a link with genuine patients? (LI 5)
		I think I need to just do more of it in order to feel more comfortable with the technology and concept (HI 41)

## Discussion

In this study we found that in line with the chronology of the COVID-19 pandemic, most treatment sessions were delivered face-to-face until the third week of March 2020 when national ‘lockdown’ restrictions were announced in the UK. This precipitated a complete stop to face-to-face sessions, a sharp rise in telephone-based sessions which was maintained throughout the study, and a slow but steady rise in video-based sessions, starting approximately 6 weeks after ‘lockdown’. We sent a survey to all clinicians in two services in July and August 2020; most respondents were conducting some video sessions, half of the respondents had conducted five or fewer sessions, although a quarter had conducted 10 or more. Overall, the LI therapist respondents had conducted fewer video-sessions than the HI therapist respondents. Although there was no evidence for differences in the proportions of HI or LI therapists reporting good experiences of using video sessions, feeling confident in delivering sessions via video, or that considered video to be an acceptable format for delivering care.

Both LI and HI respondents noted a number of barriers and frustrations using video sessions, in particular to do with the platforms through which video sessions were delivered, and problems with hardware, software or with the internet connection for themselves or their clients. It is noteworthy that national and local policy demanded changes to the platforms used, particularly due to data security concerns, and the local NHS Trusts and partner organisations advised and supported clinicians to use platforms embedded within the electronic patient record system. It may have been the case that the concerns raised by clinicians over the changes to platforms were in the early stage of such switches and some concerns may have eased after the initial problems were noted and resolved. Others noted that video sessions are more tiring, that they found them to be less efficient, and that they had difficulties with some therapeutic techniques for example using ‘stooges’ in behavioural experiments. That notwithstanding, there were many strong endorsements for using video-based therapy, with improvements in access and flexibility noted by many respondents, better attendance by clients, and a better work–life balance. In addition, many respondents found video sessions helped improve efficiency by having the ability to make notes in real-time or share resources with clients via email very quickly, and as there was no time needed to travel between locations for their sessions. Others noted improvements in the generalisability of behavioural work as experiments could be conducted via video in the clients’ own home, and in particular, many respondents commented on the better therapeutic relationship and delivery of therapy via video compared with doing so over the telephone. Overall the level of positivity about the transition to virtual treatment was similar to that of a group of psychiatrists in the USA (Uscher-Pines *et al*., 2020).

Respondents noted a number of training needs that could improve their delivery of video-based psychological treatments which are important to consider. Suggestions included the use of guidance documents, and video-tutorials to aid the learning of functions within each video platform, and troubleshoot problems that arise in sessions. Others wanted technical support in drop-in or one-to-one sessions, and a number of others proposed having time to role-play and practise using video platforms with colleagues. It may be important to address these training and development needs with service managers given the potential to optimise care in a delivery mode that might be the norm for the short-to-medium term, and may also be requested by patients or clinicians over the longer term, as a more accessible and flexible treatment option. Advice on how to deliver training, and support and supervise clinicians conducting therapy sessions via video was recently published based on learning and expertise from services that were doing so prior to the pandemic (Cromarty *et al*., 2020). It may be the case that such advice has led to changes in policy and local practices in services such as those that provided data here, and as such, some of the training needs may have been met already, or plans may be in place to meet them.

### Strengths and limitations

This study was conducted in two high-volume primary care/community based psychological therapies services that offer treatment to over 11,000 patients a year. The care delivered in these services is structured in much the same way as other Improving Access to Psychological Therapies (IAPT) services. As the lockdown which precipitated the need to deliver some sessions via video was applicable nationwide, the issues highlighted by clinicians in this study may be similar to those experienced by clinicians in other services across England. Furthermore, the findings of this study may be transferrable to other services that have yet to introduce video-based sessions. As the pandemic has surged again in many parts of the world, it is likely that further local or national lockdowns or other restrictions which would interrupt face-to-face therapy, will be a feature of at least the start, if not much of 2021 (Scudellari, 2020). However, there were a number of important limitations to this study. Firstly, data came from two services which are similarly located and any area-specific differences in restrictions due to COVID-19 could not be considered here. Secondly, the response rate for the survey was low, although this is in keeping with many online staff surveys (Preece *et al*., 2010; Rogelberg *et al*., 2006), and fewer than half of HI therapists and just over a third of LI therapists completed the survey. Although the aim of such surveys is not to necessarily achieve an entirely representative sample, this will have introduced selection biases which may have affected our results and the generalisability of them, particularly as non-responders may have had more negative experiences or more negative opinions of delivering care via video (Greenhalgh *et al*., 2017; Patel *et al*., 2020; Sayal *et al*., 2019). The information provided by respondents was informative, but we cannot be sure that we captured the breadth of experiences of clinicians working in the services, and as there was no neutral response option on the survey questions, we may not have adequately represented the views of those that held neutral opinions. However, it is noteworthy that there were very few new themes and sub-themes emerging from the final five participants. This might suggest that saturation was reached. We collected data on clinician’s experiences via a survey only and questions were open-ended, but we may have had richer data with one-to-one qualitative interviews. The time required for each clinician to complete such an interview would have made them impractical and would likely have greatly affected the number of respondents. Nonetheless, it is certainly the case that there are important questions that could not be answered by this study due to a lack of data. For example, we did not collect data on the ease or difficulty of recording sessions remotely via video or telephone, the opportunities or difficulties engaging significant others in the therapy sessions, balanced against any concerns over privacy, data security, and risk of harm from others online, and how experiences changed throughout the pandemic (Cromarty *et al*., 2020; Patel *et al*., 2020; Sayal *et al*., 2019). Furthermore, as the surveys were anonymous, no data were collected on the levels of experience of the respondents aside from their main job role, including their previous experience with remote delivered therapy, nor were data collected on the specific subtypes of therapy offered in the IAPT services as some are only offered by a few specially trained staff.

### Implications and conclusions

Video-therapy is emerging as an important mode of delivery for psychological treatment; the uptake has increased greatly in response to COVID-19, but many more sessions are still delivered by telephone. Given the likely continued demand for social distancing and restriction this puts on face-to-face therapy, and the apparent benefits noted by clinicians in delivering sessions via video compared with telephone, the barriers to delivering care this way need to be considered with some urgency as referrals for psychological therapies are expected to increase and there is likely to be continued demand for services to offer some degree of video-therapy (Holmes *et al*., 2020; Shevlin *et al*., 2020). However, the limited previous research suggesting similar efficacy of video versus face-to-face therapy (Day and Schneider, 2002; Stubbings *et al*., 2013) needs to be further validated in mental health services, especially as specific patient characteristics have been associated with lower uptake (Eberly *et al*., 2020), and there is potential that certain patient groups may be under-served by this mode of treatment.

Should the findings of this study be replicated and confirmed in larger samples and a broader range of settings, we might suggest that services need to quickly understand the deficiencies and training needs for their staff and patients, so that they are prepared to continue offering psychological therapies without it affecting the volume or quality of care they are able to deliver. There are informative advisory manuscripts that offer guidance to do this based on years of experience in delivering care this way (Cromarty *et al*., 2020; Freeston *et al*., 2020). It can be expected that a number of patients and clinicians may have insufficient hardware or internet connections to support the use of video sessions. In such instances, services and the organisations operating them might provide hardware on a temporary basis for their patients or staff to use for their sessions. Such hardware might include tablets or laptop computers, and WiFi signal boosters to improve their internet connections. Issues with the platforms on which video-based sessions are delivered also need addressing. This might involve working with providers of these platforms to support them in making adjustments to them where necessary, and might include training and drop-in or support sessions with clinicians that lack confidence in using them, or lack knowledge in the specific functions that facilitate the delivery of treatment. This would help ensure the benefits of video-based sessions and access to care are not limited to only a few patients. Further training needs have to be addressed, and both video tutorials and best practice guides, specific to the local service context, need to be developed and shared with clinicians as a top priority. Services might also consider how to provide (additional) reflective spaces, in order to support staff feeling overwhelmed or struggling with the changes being made to the delivery of care, or related to the wider context in which such changes are necessitated. In addition, particular focus might be given to training or supporting LI therapists to increase their use of video sessions, where appropriate, and if their clients want to have sessions in such a way. The majority of LI sessions pre-COVID-19 were conducted over the telephone, which may be preferred by both clients and clinicians, so it was expected that there would be differences in the uptake of video therapy across staff teams with LI and HI therapists. Future research might investigate potential differences in the advantages of delivering therapy via video compared with doing so over the telephone or face-to-face with both LI compared with HI therapies, and in sessions that typically last 30 minutes compared with those that typically last 50–60 minutes. It would also be informative to compare the experiences of service users and clinicians. Future trials might also address the question of whether there are different patient outcomes on average, or for particular sub-groups of people engaging in psychological therapy via video, telephone, or face-to-face. Such research may then inform personalised care and person-centred joint decision making.

In addition to changes made by services, clinicians should take the time to learn the different functions of the platforms used to deliver video sessions, and go through troubleshooting guides and tutorials to support their clients with technological problems as needed. It may be important to share positive experiences of video therapy with peers (if experiences are positive) as it seems that a number of respondents were not aware that many of their peers liked video sessions and found that they went well, but instead were put off trying to use video sessions by the apparent negative experiences of their peers. Clinicians might organise a timetable of when they are available to support their peers conducting behavioural work including stooge experiments for patients with social phobia. Sharing of top tips and best practice based on what has been learned by those that have conducted many sessions via video may also prove helpful and provide encouragement for clinicians feeling less confident conducting video sessions.

## Data Availability

The authors confirm that the qualitative data supporting the findings of this study are available within the article; further data from the survey used to collect data may be available from the corresponding author upon reasonable request. Restrictions apply to the availability of the quantitative data on individual service users which were used under licence for this study. These data are available from the corresponding author subject to permission from Camden & Islington NHS Foundation Trust and with the appropriate ethical approval.

## Supplementary material

To view supplementary material for this article, please visit: https://doi.org/10.1017/S1352465821000187
